# The Gerontological Case Against Fossil Fuels

**DOI:** 10.1093/geroni/igab045

**Published:** 2021-10-22

**Authors:** Kathy Sykes

**Affiliations:** U.S. Environmental Protection Agency, Washington, District of Columbia, USA (Retired)

**Keywords:** Climate change, Extreme weather events, Health impact and risks

## Abstract

Climate change is a threat to persons of all ages, but the older population is especially susceptible to the harms of extreme heat, growing air toxics, increasingly prevalent extreme weather events, and related environmental hazards. These changes reflect our continuing dependence on fossil fuels. The effects range from increased risk of chronic conditions to disruption of essential medical and social supports. We need the expertise of gerontologists to craft practical and evidence-based mitigation and adaptation interventions for climate change if we are to meet the needs of aging populations. We cannot wait for others to raise their voices. Gerontologists must address the challenge of climate change and support energy policies that terminate the fossil fuel supply chain.

**Translational Significance:** To respond to the threat of climate change, we must support policies and promote investment in renewable energy sources, not fossil fuels, for transportation, energy-efficient buildings, and housing and power generation. Gerontologists can play a key role in developing effective science-based strategies to mitigate the impact of climate change.

While climate change affects all of us, not enough attention has been paid to its impact on the health and well-being of older adults. If we are to focus greater attention on the needs of the older population, we need gerontologists to become part of the solution.

In 1896, the Swedish scientist Svante Arrhenius was the first to calculate estimates of the extent to which increases in atmospheric carbon dioxide (CO_2_) would increase Earth’s surface temperature through the greenhouse effect (1). Arrhenius knew that CO_2_ absorbs radiation, felt by us as a form of heat. He also knew that the Industrial Revolution’s steam engines that powered ships and industries burned lots of coal—and that burning coal produced large quantities of CO_2_. Arrhenius wondered whether doubling CO_2_ concentration in the atmosphere would increase the Earth’s surface temperature (1).

Another Swede, 15-year-old Greta Thunberg, a distant relative of Arrhenius, became the face of climate activism when she skipped school on Fridays and protested in front of the Swedish Parliament on its inaction on climate change. Thunberg’s *Fridays for Future* school strike has spread all over the world.

At last year’s Davos World Economic Forum, Thunberg said, “No political ideology or economic structure has been able to tackle the climate and environmental emergency and create a cohesive and sustainable world . . . . because that world, if you have not noticed, is on fire” (2).

Greta is right; we absolutely need the political will to save the Earth. And we all must raise our voices and demand action now. Gerontologists need to add their voices to the chorus for change.

My hope is that by laying out the threats of climate change and its impact on the health of older adults, I can move you to action. We need political action—educating elected officials to make the right choices.

## The Impact on Health of Older Adults

When I worked at the US Environmental Protection Agency, I was a member of the Interagency Forum on Aging-Related Statistics and helped develop an indicator on environmental health. This air quality indicator debuted in the 2004 edition of the Forum’s chartbook and represented the percentage of persons 65 and older living in counties with measured air pollutant concentrations above the level of the national public health standards. In 2018, approximately 137 million people lived in counties where monitored air was unhealthy at times due to high levels of at least one of the 6 principal air pollutants: ozone, particulate matter (PM), nitrogen dioxide (NO_2_), sulfur dioxide, carbon monoxide, and lead. About 12%, or nearly 16 million people, of those living in counties where monitored air quality was unhealthy at times were aged 65 and older. Unhealthy air in the vast majority of counties was linked to one or both of 2 pollutants—ozone and PM_2.5_ (3).

A recent study found minorities breathe more PM_2.5_ on average, a finding that holds across income levels and regions of the United States. The finding contributes to a growing body of evidence showing that African Americans, Hispanics, Asians, and other people of color are disproportionately exposed to air pollution (4). The study did not examine age—a ripe topic for research.

Through extensive research, we know that air pollution can aggravate chronic conditions such as chronic heart and lung diseases, common in older adults, and lead to more health care visits, increased medication usage and emergency room and hospital stays, and even death. Researchers have also found that particle pollution can cause intestinal diseases—appendicitis, colorectal cancer, and inflammatory bowel disease (5,6). Furthermore, there is some evidence that PM_2.5_ and NO_2_ can trigger the spread and lethality of the coronavirus disease 2019 (7). Long-term exposure to air pollution is linked to increasing the risk for dementia. Recent research suggests short-term exposure to PM_2.5_, even at “acceptable levels,” may impair mental ability in older adults (8).

## Wildfires, Particle Pollution: Threats to Older Americans

Wildfires worldwide are increasing in number, size, and severity. In the United States, they produce about a third of the country’s PM. Prolonged stretches of record high temperatures associated with droughts are contributing to dry conditions that promote wildfires.

This year, firefighters have wrapped the base of the world’s largest tree, General Sherman (as well as other sequoias), in a fire-resistant blanket in an effort to save it from wildfires burning in California’s Sequoia National Park. While giant sequoias are adapted to fires, climate change has made fighting fires more challenging, and these extremely hot wildfires can overwhelm the trees (9,10).

Population aging and growing numbers of wildfires place a greater challenge on protecting older people from exposure to wildfire smoke. Issues regarding mobility and warning systems make wildfires especially challenging.

Wildfires can also cause physical injury, psychological stress and smoke-related illness, and death. A recent study examined California wildfires over 2 decades to see whether burned areas and fire frequency differed across Census tracts according to socioeconomic indicators. They found the number of Census tracts that experienced major wildfires had doubled between 2000 and 2020 in the number of people residing in wildfire-impacted Census tracts. They attributed the increase to more than a 23 000-acre/year increase in the area burned by wildfires. Census tracts with a higher fire frequency and burned area had lower proportions of minority groups on average, with one exception—Native American populations. Moreover, high-impact Census tracts had “higher proportions of low-income residents. They also had a higher proportion of older residents” (11).

While the body of scientific literature on the threats posed by climate change and smoke from wildfires continues to grow, more research is needed to determine the best strategies for protecting public health of at-risk populations. The pros and cons must be weighed for an evacuation versus sheltering in place with high efficiency particulate air filters to minimize morbidity and mortality, especially for persons living with respiratory and or cardiopulmonary illnesses. The growing threats from wildfire smoke must be matched with scientific guidance to address the increasing number of older persons and increasing intensity and length of the wildfire season. We need to develop better wildfire smoke forecasting methods to ensure that professionals in the fields of aging and public health are informed on effective strategies to reduce exposure to those at greatest risk.

## Economic Toll and Breaking Records

The economic toll of climate change is catastrophic. According to National Oceanic and Atmospheric Administration, 2020 was the sixth consecutive year (2015–2020) in which 10 or more billion-dollar weather and climate disaster events have affected the United States. And we keep breaking records and the hearts of families of the victims of these billion-dollar disasters. This year could be even more catastrophic than previous years. June 2021was the hottest ever recorded in the United States, and July was the hottest on record worldwide. Almost half of the lower 48 states are currently experiencing drought conditions, and scientists warn that if we do not reduce greenhouse gas emissions, things will become much worse (12).

This year, the International Panel on Climate Change 6 reported climate disasters are increasing both in cost and numbers due to a combination of greater exposure (ie, more assets at risk), vulnerability (ie, how much damage a hazard of given intensity—wind velocity, or flood depth), and the frequency of events that result in billion-dollar disasters (13). The report stated that over the past 20 years, heat-related mortality among people 65 and older had increased by more than 50% (14). They also said that no temperature rise is “safe.” Even if countries sharply reduced emissions, “total global warming is likely to rise around 1.5 degrees Celsius within the next two decades, a hotter future that is now essentially locked in” (15).

## Extreme Heat

Each year, more people die (600) from extreme heat in the United States than any other weather event (16). Once again, the older population is at greater risk compared to other age cohorts. Older adults have underlying health conditions, such as heart, kidney, or respiratory disease, that make them more vulnerable to heat. Moreover, certain medications (eg, blood pressure medications) can reduce the body’s ability to sweat.

When the body overheats and becomes dehydrated, the blood thickens. As a result, the heart pumps harder, which can seriously damage it and other organs. Mechanisms that allow the body to respond to excessive heat—especially sweating—can fail if humidity is high, which prevents the evaporation of perspiration.

A recent study found older persons experienced the highest mortality rate—40%—from heat exposure of all age groups. The authors posited that those at greater risk might be more susceptible to heat stress due to their inability to regulate their body temperature and/or due to social isolation (17). These numbers of older adults suffering from heat stress or death are expected to increase due to climate change; however, these deaths are preventable. Gerontologists with an emphasis on public health could develop a local early warning and response system for the older population, identifying those with certain health conditions, lacking air conditioning, or those who are socially isolated and/or poor.

Climate change’s impact on health requires more research on adaptation, mitigation, supportive services, and effective communication strategies to ensure those at greater risk are protected. Emergency responders need effective science-based strategies to meet the needs of older persons.

## Ending Subsidies

To respond to the threat of climate change, we must support policies and promote investment in renewable energy sources, not fossil fuels, for transportation, energy-efficient buildings and housing, and power generation. For many decades, the United States has supported the production of inexpensive fossil fuels including coal, oil, and natural gas. These energy markets are generally highly profitable and given their deleterious impact on climate change and health, and the existence of renewable alternatives, these subsidies should cease immediately (18).

President Biden’s Build Back Better plan addresses the climate crisis by creating jobs that pay well, establishing standards for energy efficiency and clean energy increasing a tax credit for electric vehicles, and creating a new Civilian Climate Corps (19). The price to chart a new course and reach emission reductions by 2030 is $3.5 trillion. Sounds expensive, but as the columnist E. J. Dionne noted, that investment is spread over the next 10 years. By comparison, the US gross domestic product is expected to be $288 trillion, so the cost would be 1.2% of our economy (20). It seems to be a good bargain to save humanity and the Earth. But at this writing, its enactment is uncertain.

## A Call to Action

In September 2021, leading public health journals copublished an editorial that climate change is the “greatest threat” to global public health and that world leaders must act now to cut greenhouse gas emissions to avoid “catastrophic harm to health that will be impossible to reverse.” This unprecedented joint statement by more than 200 journals, including *The Lancet*, *The New England Journal of Medicine*, and GSA’s *The Gerontologist*, *The Journals of Gerontology, Series A: Biological Sciences and Medical Sciences*, and *Innovation in Aging*. There is still an opportunity to stop climate change from becoming much worse (21). Indeed, we can bend this arc of inevitable disaster if we all make it our work to address climate change to benefit every generation. We have the tools. Now we need the will.
